# Evaluating the Window Size’s Role in Automatic EEG Epilepsy Detection

**DOI:** 10.3390/s22239233

**Published:** 2022-11-27

**Authors:** Vasileios Christou, Andreas Miltiadous, Ioannis Tsoulos, Evaggelos Karvounis, Katerina D. Tzimourta, Markos G. Tsipouras, Nikolaos Anastasopoulos, Alexandros T. Tzallas, Nikolaos Giannakeas

**Affiliations:** 1 Department of Informatics and Telecommunications, University of Ioannina, 47100 Arta, Greece; 2 Department of Electrical and Computer Engineering, Faculty of Engineering, University of Western Macedonia, 50100 Kozani, Greece; 3 Department of Electrical and Computer Engineering, University of Patras, 26504 Rio, Greece

**Keywords:** EEG, seizure detection, window size, neural network, genetic algorithm, k-nearest neighbours

## Abstract

Electroencephalography is one of the most commonly used methods for extracting information about the brain’s condition and can be used for diagnosing epilepsy. The EEG signal’s wave shape contains vital information about the brain’s state, which can be challenging to analyse and interpret by a human observer. Moreover, the characteristic waveforms of epilepsy (sharp waves, spikes) can occur randomly through time. Considering all the above reasons, automatic EEG signal extraction and analysis using computers can significantly impact the successful diagnosis of epilepsy. This research explores the impact of different window sizes on EEG signals’ classification accuracy using four machine learning classifiers. The machine learning methods included a neural network with ten hidden nodes trained using three different training algorithms and the k-nearest neighbours classifier. The neural network training methods included the Broyden–Fletcher–Goldfarb–Shanno algorithm, the multistart method for global optimization problems, and a genetic algorithm. The current research utilized the University of Bonn dataset containing EEG data, divided into epochs having 50% overlap and window lengths ranging from 1 to 24 s. Then, statistical and spectral features were extracted and used to train the above four classifiers. The outcome from the above experiments showed that large window sizes with a length of about 21 s could positively impact the classification accuracy between the compared methods.

## 1. Introduction

Epilepsy is the most common condition affecting the central nervous system, where 80% of the patients are citizens from developing or middle-income countries [1]. Besides the young population, it can also occur in the elderly population (people over 65 years old) [2]. Epilepsy has a severe economic impact in terms of healthcare needs. It causes premature deaths and can lead to lost work productivity. Considering all the above reasons, it is an essential topic in the biomedical sciences [1,3].

Epilepsy is a chronic brain disease characterized by seizures affecting all age groups. It causes recurrent seizures, ranging from one episode per year to several episodes per day. There is a distinction between epilepsy and seizures since not all seizures are epileptic fits. The main characteristic of epilepsy is that it is responsible for triggering unprovoked recurrent seizures caused by chronic abnormal bursts of electrical discharges in the brain [4]. This process is called “epileptogenesis” and makes epilepsy highly unpredictable. Other types of seizure disorders can be activated by various causes, which can be measured, including stroke, tumours, and other space-occupying lesions. Secondary or symptomatic epilepsy is epilepsy caused due to an underlying abnormality of the structure of the brain and is the type of epilepsy where preventive measures can be applied according to various causes. It can be noted that more than 60% of the cases lack a definitive cause. This epilepsy type is called primary or idiopathic epilepsy and is not preventable but can be treated using antiepileptic medicines [3,5].

The occurrence of epileptic seizures is due to a malfunction in the brain, which triggers a sudden excessive electrical discharge in a group of cells in the brain’s cerebral cortex. This malfunction causes motor function abnormalities, resulting in tonic–clonic muscle spasms. The vast and abrupt energy surge triggered by the brain’s neurons is the cause of epileptic seizures, which show differences in their properties. Seizures range from a few seconds to severe, generalized, and prolonged convulsions, leading to dangerous and life-threatening situations. Seizures’ characteristics depend on the specific brain region involved, the extent of the abnormal electrical discharge and its spread [3,6].

The limited knowledge regarding the human brain creates a challenge in understanding the properties of a brain with epilepsy. The disease’s temporary symptoms include mindfulness loss, minor (almost undetectable) abnormalities in movement, mild muscle twitching, and abnormalities in visual, auditory, and gustatory senses and mood. The epileptic seizures start and finish unexpectedly, without involving interference from the external environment, and it is possible to remain unnoticed. For this reason, detecting and measuring epileptic seizures is a challenging task [3,7].

Seizure occurrence is not always connected to epilepsy since, statistically, 10% of the world population will have one seizure during their life [3]. These nonepileptic seizure types can be caused by chemical imbalances. If two or more seizures occur without a specific reason, it may have been caused due to epilepsy. In case of epileptic seizures, the patient can start receiving antiepileptic medicines to improve their safety and quality of life. The unpredictable nature of epileptic seizures can be a severe life-threatening cause (e.g., if they are triggered while driving a car or swimming). The most common method for diagnosing epilepsy is an electroencephalogram (EEG) signal analysis. EEG signals reflect the brain’s electrical activity at a given timestamp [3].

An EEG can record the electrical brain activity using a series of electrodes placed on the patient’s scalp. Brain abnormalities that are not related to epilepsy can be analysed by studying EEG signals. Soikkeli et al. [8] investigated the generalized slowing of the EEG in patients with Parkinson’s disease. Wieser et al. [9] studied Creutzfeldt–Jakob disease using EEG signals while Neto et al. [10] conducted a regularized linear discriminant analysis of EEG features taken from patients with dementia [3]. Overall, EEG has been used for the detection and quantification of many neurological diseases [11] or conditions [12] or cognitive states such as stress induction [13,14], thus becoming a significant tool for neurologists.

The study of epileptic seizures analyses EEG signals received before and during the seizures, which contain patterns that differentiate them from those recorded in a nonepileptic person. The identification of epileptic seizures is made by observing the EEG data. For this reason, an EEG signal analysis approach which provides information regarding the brain’s condition must be applied [3].

This paper explores the impact of the window size on classifying epileptic short-term EEG signals using four machine learning methods. The machine learning methods used were a single-layer neural network (SLNN) with ten hidden nodes, trained using three different training algorithms, and the k-nearest neighbours (K-NN) classifier [15]. The neural network training methods were the Broyden–Fletcher–Goldfarb–Shanno (BFGS) algorithm [16], the multistart algorithm for global optimization problems proposed by [17], and the modified genetic algorithm (GA) proposed by Tsoulos [18].

This paper is structured into six main sections, starting with an “Introduction”, which explains the significance of epilepsy, the importance of EEG for its diagnosis, and includes a short description of the research’s motivation. The “Related Work” section contains existing work regarding automated methods for diagnosing epilepsy. The “Methods” section presents four machine learning methods for exploring the window size’s effect on classifying epileptic short-term EEG signals. The “Results” section analyses the four machine learning algorithms’ results presented above using different window types applied to the University of Bonn epilepsy database [19]. The following two sections contain the “Discussion” and “Conclusion”. Finally, the “Methods” section describes each machine learning method used to explore the window size effect on classifying epileptic short-term EEG signals.

## 2. Related Work

Existing seizure detection works include the method proposed by Naghsh-Nilchi and Aghashahi [20]. The proposed approach was based on two eigensystem pseudospectral estimation methods: eigenvector and multiple signal classification for time-domain EEG signal pseudospectrum estimation. The pseudospectrum was partitioned into sub-bands, each having a smaller frequency. Then, a feature extraction stage was applied to produce the input to a multilayer perceptron (MLP). The MLP classified the input vectors into three classes: normal, interictal and ictal. Tzallas et al. [21] compared various time–frequency (t-f) analysis methods for categorizing epileptic seizures EEG segments. A three-stage analysis was utilized, starting with the t-f analysis and a power spectrum density (PSD) calculation from each EEG segment. The next stage involved the extraction of a feature set by measuring the signal segment fractional energy on specific t-f windows. In contrast, the third stage was the categorization (normal and epileptic) of the EEG segment using artificial neural networks (ANNs). Martinez-del Rincon et al. [22] used an EEG analysis system for automatic epilepsy seizure detection that could exploit EEG data’s underlying nonlinear nature. Hassan and Subasi [23] addresses the automated seizure detection problem using single-channel EEG signals. The EEG signal segments were initially decomposed using the complete ensemble empirical mode decomposition with adaptive noise (CEEMDAN) signal processing model. The training and testing data were formed by extracting six spectral moments from the CEEMDAN mode functions, which were entered as inputs to the linear programming boosting (LPBoost) classifier. Juarez-Guerra et al. [24] used a wavelet analysis system for identifying epilepsy seizures from EEG signals. The proposed system utilized the discrete wavelet transform (DWT) and the maximal overlap discrete wavelet transform (MODWT) for extracting a feature set. This set was entered as input to an ANN, which performed the classification task. Hossain et al. [25] used a CNN for feature learning from raw EEG data to detect seizures on an open-access EEG epilepsy dataset from the Boston Children’s Hospital [26]. The proposed model extracted spectral and temporal features from EEG epilepsy data and utilized them to learn the overall structure of a seizure that was less sensitive to variations. Nicolaou and Georgiou [27] explored the use of permutation entropy (PE) as a feature for automatic epilepsy seizure detection. Their method utilized a support vector machine (SVM) for the binary classification task and was based upon the observation that the PE dropped during a seizure. Shoeb and Guttag [28] presented a method utilizing an SVM to construct patient-specific classifiers that could use EEG signals from patients’ scalps to detect the onset of epileptic seizures. Guo et al. [29] proposed an EEG-based method for automatic epileptic seizure detection, which utilized the approximate entropy features derived from the multiwavelet transform. These features were introduced as input data to an ANN for classifying the EEG signals as epileptic or nonepileptic. Subasi [30] decomposed EEG signals into their frequency sub-bands using a wavelet transform. Then, these sub-bands were introduced as input to an ANN for classification into two categories (epileptic and nonepileptic). Moreover, this research developed and compared classifiers based on feedforward error backpropagation ANNs and dynamic wavelet networks. The comparison was made to test their accuracy in EEG signals classification. Ghosh-Dastidar et al. [31] combined the mixed-band wavelet-chaos methodology [32,33] with a principal component analysis (PCA)-enhanced cosine radial basis function neural network classifier for classifying EEG signals into three categories (healthy, ictal, and interictal). Guo et al. [34] proposed a method for automatic epileptic seizure detection. This method utilized line length features based on a wavelet transform multiresolution decomposition and introduced them as input to an ANN for classifying the EEG signals into two categories (healthy or epileptic). Hassan et al. [35] proposed an automated epilepsy diagnosis system based on a tuneable-Q factor wavelet transform and bootstrap aggregating. Finally, the general-purpose method proposed by Tsoulos et al. [36] utilized genetic programming to create ANNs. The proposed method could infer the ANN’s architecture and estimate the optimal number of neurons for each given problem.

## 3. Materials and Methods

This research studied the four machine learning methods that are analysed in the Methods section for exploring window size’s effect on classifying epileptic short-term EEG signals.

The well-established epileptic database from the University of Bonn was used for the evaluation, since it is the most used database from the published databases. The Bonn database consists of 5 groups of recordings namely Z-O-N-F-S. The Z and O datasets consist of EEG recordings of healthy, nonepileptic participants with closed and open eyes, respectively. The N, F, and S subsets include intracranial EEG recordings acquired from five epileptic patients, during presurgical examination. Specifically, the N subset includes parts of interictal recordings originating from the epileptic zone of the opposite hemisphere, while the O subset includes parts of EEG recordings obtained from the epileptic zone. The S subset includes 100 intracranial EEG recordings, obtained from the epileptogenic zone during epileptic activity. The epileptogenic zone was the hippocampus and no further patient data were provided.

For the classification task, all 5 subsets of the Bonn database were used, for a 5-class Z-O-N-F-S problem. Each group consisted of 100 single-channel recordings with 23.6 s duration and all recordings were used for the training and testing. Before the experiment, a low-pass FIR filter at 40 Hz was applied to all recordings, and then the recordings were split into datasets of different time window lengths. The examined window lengths were 1–24 s (24 s being in fact 23.6 s).

For each examined window length, a set of extracted univariate and spectral features were calculated to create a feature vector. Specifically, the following time-domain features were extracted: mean, median, variance. Moreover, a fast Fourier transform was employed to transform the signal into the frequency domain and the spectrum amplitude of four EEG bands was calculated. The EEG bands were:

- Alpha band (8–12 Hz)
- Beta band (12–25 Hz)
- Theta band (4–8 Hz)
- Delta band (1–4 Hz)

The following subsections analyse the machine learning methodologies that were tested for the classification of the 5-class problem and the evaluation of the time window length. Particularly, Section 3.1, Section 3.2 and Section 3.3 analyse the optimization techniques used to optimize the hyperparameters of a 10-layer multilayer perceptron neural network. Section 3.4 analyses the last classification methodology, k-nearest neighbours (kNN).

### 3.1. The BFGS Method

The BFGS algorithm is a quasi-Newton approach utilizing a new updating formula which has become very popular and has been subjected to numerous modifications. Quasi-Newton methods are used to solve unconstrained optimization problems [16,37,38,39,40,41].

An unconstrained optimization problem can be described by using Equation (1):

(1) $$\min_{x\in R^{n}}f\left(x\right)$$

In this formula, $R^{n}$ denotes an n-dimensional Euclidean space while $f:R^{n}\to R$ is continuously twice differentiable. The update formula of BFGS is defined in Equation (2) where $s_{k}$ and $y_{k}$ are the step vectors, and *g* is used to denote the gradient for Equation (1).

(2) $$\begin{array}{ccc} s_{k} & \overset{def}{=} & x_{k+1}-x_{k}\\ y_{k} & = & g_{k+1}-g_{k} \end{array}$$

The BFGS method is considered the best among all quasi-Newton based methods. The updating formula for BFGS takes the form shown in Equation (3).

(3) $$B_{k+1}=B_{k}+\frac{y_{k}y_{k}^{T}}{y_{k}^{T}s_{k}}-\frac{B_{k}s_{k}s_{k}^{T}B_{k}}{s_{k}^{T}B_{k}s_{k}}$$

In this formula, the $B_{k}$ symbol denotes the Hessian approximation at $x_{k}$, and the matrix $B_{k+1}$ is generated by (3) to satisfy the following secant formula:

(4) $$B_{k+1}s_{k}=y_{k}$$

The above secant formula is considered an approximation of the Newton relation. The secant can be fulfilled if $s_{k}^{T}y_{k}>0$, which is called the curvature condition and ensures that the BFGS updating matrix shown in (3) is positive definite [16]. Unconstrained optimization problems are solved using an iterative procedure. Equation (5) defines the iterative formula for quasi-Newton methods.

(5) $$x_{k+1}=x_{k}+a_{k}d_{k}$$

In this formula, the term $a_{k}$ defines the step size while $d_{k}$ defines the search direction. The step must be a positive number in order f(x) to be able to reduce sufficiently, while both $a_{k}$ and $d_{k}$ must be chosen carefully for an efficient search line. The step size can be calculated by using various formulas divided into two main categories (exact or inexact line search). An ideal choice would be the exact line choice defined by the formula $a_{k}=\arg\min\left(f\left(x_{k}+a_{k}d_{k}\right)\right),a>0$ but it is computationally intensive to define this value. The reason behind this problem is that it requires a large number of evaluations for the objective function *f* and its gradient *g*. The inexact line search has a number of formulas proposed by different researchers, including the formulas of Armijo [42], Wolfe [43,44], and Goldstein [45] with the first one being the easiest one to implement. The Armijo search line formula is defined in (6).

(6) $$f\left(x_{k}\right)-f\left(x_{k}+a_{k}d_{k}\right)\geq -\sigma a_{k}g_{k}^{T}d_{k}$$

Given s>0,λ∈(0,1),σ∈(0,1) and $a_{i}=\max\left\{s,s\lambda ,s\lambda^{2},\ldots \right\}$ such that k=0,1,2,3,…, the reduction in *f* should be proportional to both the step size and directional derivative $g_{k}^{T}d_{k}$ [16].

The search directions are important for determining the *f* value, and the quasi-Newton methods can be defined using the following equation.

(7) $$d_{k}=-B_{k}^{-1}g_{k}$$

In this formula, $B_{k}$ is a nonsingular symmetric approximation matrix of the Hessian defined in (3). The initial matrix $B_{0}$ is an identity matrix updated by an update formula. When $d_{1}$ is defined from the above formula and $B_{k}$ is a positive definite matrix, then $d_{k}^{T}=-g_{k}^{T}B_{k}^{-1}g_{k}<0$, which makes $d_{k}$ a descent direction. Algorithm 1 describes the iterative process of the BFGS algorithm [16].
**Algorithm 1**: The BFGS Algorithm  1: Having a starting point $x_{0}$ and $B_{0}=I_{n}$. Set the values for s,β, and σ.  2: End if $∥g(x_{k+1})∥<10^{-6}$.  3: Calculate the search direction using Formula (7).  4: Calculate the difference $s_{k}=x_{k+1}-x_{k}$ and $y_{k}+g_{k+1}-g_{k}$.  5: Update $B_{k}$ by (3) in order to obtain $B_{k+1}$.  6: k=k+1.  7: Go to step 2.

The current research uses the BFGS variant proposed by Powell [46]. The main advantage of Powell’s methodology is that the step along the search direction is not restricted by constraints having small residuals, which significantly increases efficiency, specifically the nearly degenerate constraints.

### 3.2. The Multistart Method

The multistart method described in Algorithm 2 is a two-phase stochastic black-box global optimization approach consisting of a global and a local phase. In black-box optimization problems, no known structure can be used, and the problem can be formulated by minimizing, for example, a continuous function *f* over a compact set $S\subseteq R^{n}$. Due to the nature of stochastic problems where the outcome is random, it is particularly suitable for black-box optimization problems. Another characteristic of these approaches is that they require little to no assumptions about the optimization problem. On the other hand, they can only provide a probabilistic convergence guarantee in the best-case scenario [47].

In the first phase of a two-phase method, many randomly sampled points in the feasible region are used to evaluate the function. In the second phase, a local search procedure is applied to each sample point mentioned above, yielding various local optima. Amongst all local optima, the best one forms the resulting estimation of the global optimum [17,47].
**Algorithm 2**: The Multistart Algorithm  1:   i=0 and $X^{*}=$.  2:   Take a random sample *x* from *S*.  3:   Start a deterministic local search process at *x* and conclude at a local minimum $x^{*}$.  4:   Check if a new minimum is found.  5:   $x^{*}\notin X^{*}$ then  6:           i←i+1.  7:           $x_{i}^{*}=x^{*}$.  8:           $X^{*}\leftarrow X^{*}\cup \left\{x_{i}^{*}\right\}$.  9:   end.  10: If ending criteria have been met, terminate the process.  11: Go to step 2.

### 3.3. The Modified GA Method

GAs are global optimization methods based on Charles Darwin’s theory of natural evolution. A GA begins with a pool of candidate solutions, which are the artificial equivalent of chromosomes in biological organisms. Then, these chromosomes are evolved in an iterative process using the selection, crossover, and mutation genetic operations. The process is continued until the termination criterion is reached, or the algorithm converges to the best chromosome, which can be the optimal or a suboptimal solution of the problem [18].

The real-coded GA proposed by Kaelo and Ali [48] can be seen in Algorithm 3. In this algorithm, the problem is to find the global minimum of the following unconstrained optimization problem.

(8) minimizef(x)subjecttox∈Ω

where $f\left(x\right):\Omega \subset R^{n}\to R$ is a continuous real-valued function and *x* is an n-dimensional continuous variable vector. The term Ω denotes a box or other region which is easy to sample. The $x_{opt}$ point is the global minimizer of *f* if $f_{opt}=f\left(x_{opt}\right)\leq f\left(x\right),\forall x\in \Omega$. At each iteration of the algorithm (generation), the candidate points set *S* is updated which new chromosomes (offspring) created by the reproduction process (crossover and mutation) of the algorithm [18,48].
**Algorithm 3**: The Real-Coded GA  1:    Create *N* random points in Ω from the uniform distribution.  2:    Store the points in set *S*.  3:    iter=0.  4:    Evaluate each chromosome using its function value.  5:    If the termination criteria are achieved, stop the GA.  6:    Select m≤N parents from *S*.  7:    Create *m* offspring using the selected parent chromosomes of the previous step.  8:    Mutate the offspring with probability $p_{m}$.  9:    Remove the *m* worst chromosomes and replace them with the offspring.  10: Create a trial point $\tilde{x}$. If $f\left(\tilde{x}\right)\leq f\left(x_{h}\right)$ where $x_{h}$ is the current worst point in *S*, then replace $x_{h}$ with $\tilde{x}$.  11: iter=iter+1.  12: Go to step 4.

The real-coded GA starts by creating the initial population in the first two lines, followed by the initialization of the generation counter. The following step evaluates the population. In step 5, the GA checks if the termination criteria have been achieved and terminates the algorithm. The termination is done when $|f_{h}-f_{1}|\leq e$ or the maximum number of iterations has been reached. The term $f_{h}$ denotes the function value of the most optimal chromosome in the population, while $f_{h}$ denotes the function value of the least optimal chromosome in the population. If the termination criteria have not been achieved, the evolution process continues. In step 6, the selection of two parent chromosomes $x=(x_{1},x_{2},\ldots ,x_{n})$ and $y=(y_{1},y_{2},\ldots ,y_{n})$ for the reproduction process is done using the tournament selection [49] mechanism. Step 7, creates the offspring using the equations shown in (9)

(9) $$\begin{array}{ccc} \tilde{x_{l}} & = & a_{i}x_{i}+\left(1-a_{i}\right)y_{i}\\ \tilde{y_{l}} & = & a_{i}y_{i}+\left(1-a_{i}\right)x_{i} \end{array}$$

where $a_{i}\in \left[-0.5,1.5\right]$ [50]. The mutation procedure in step 8 follows the formula depicted in (10).

(10) $$x_{i}^{\prime }=\left\{\begin{array}{cc} x_{i}+\Delta \left(iter,r_{i}-x_{i}\right), & t=0\\ x_{i}-\Delta \left(iter,x_{i}-l_{i}\right), & t=1 \end{array}\right.$$

In this formula, *t* is a random number taking the values 0 or 1, iter is the current generation and $\Delta \left(iter,y\right)=y\left(1-r^{\left(1-\frac{iter}{ITERMAX}\right)}\right)$ with r∈[0,1] and ITERMAX being the maximum allowed number of generations. Step 9 replaces the *m* worst chromosomes in the population with the offspring. Step 10 is the local technique that creates trial points to replace the least optimal points in the population. Using the following equation, this technique initially selects a random point *y* from *S* and creates a trial point $\tilde{x_{i}}$.

(11) $$\tilde{x_{i}}=\left(1+\gamma_{i}\right)x_{l,i}-\gamma_{i}y_{i},\;i=1,\ldots ,n$$

where $\gamma_{i}\in \left[-0.5,0.5\right]$ and $x_{l,i}$ is the *i*th component of the most optimal chromosome $x_{l}$. The technique ends by replacing the least optimal point $x_{h}$ in *S* with $\tilde{x}$, if $f\left(\tilde{x}\right)\leq f\left(x_{h}\right)$ [18,48].

The current paper used the modifications proposed by Tsoulos [18]. These modifications include a novel stopping rule, a new mutation operator, and a local search procedure application. This procedure is applied to the most optimal chromosome $x_{l}$ every $K_{ls}$ generations, with $K_{ls}$ being a constant that defines the frequency of the applied local search procedure.

### 3.4. The K-NN Classifier

The K-NN algorithm is one of the simplest and oldest classification algorithms [15]. It has a set containing *n* samples $D_{n}=\left\{\left(X_{1},Y_{1}\right),\ldots ,\left(X_{n},Y_{n}\right)\right\}$, where $X_{i}\in R^{d}$ are the vectors containing the features and $Y_{i}\in \{\omega_{1}$, $\omega_{2},\ldots ,\omega_{M}\}$ are the labels which correspond to each class. The K-NN algorithm categorizes a new input pattern *x* into the class of its nearest neighbour in the *n* training examples. The identification of the closest class is made using the Euclidean distance (although other distance metrics can be used) [51,52]. The K-NN method can be seen in Algorithm 4.
**Algorithm 4**: The K-NN Algorithm  1: Classify (X,Y,x).  2: for i=1 to *n* do  3:          Calculate the Euclidean distance $d_{E}\left(X_{i},x\right)$.  4: end.  5: Compute set *I* having the indices for the *k* smallest distances $d_{E}\left(X_{i},x\right)$.  6: Return majority label for $Y_{i}$ where i∈I.

## 4. Results

**Figure 1:**
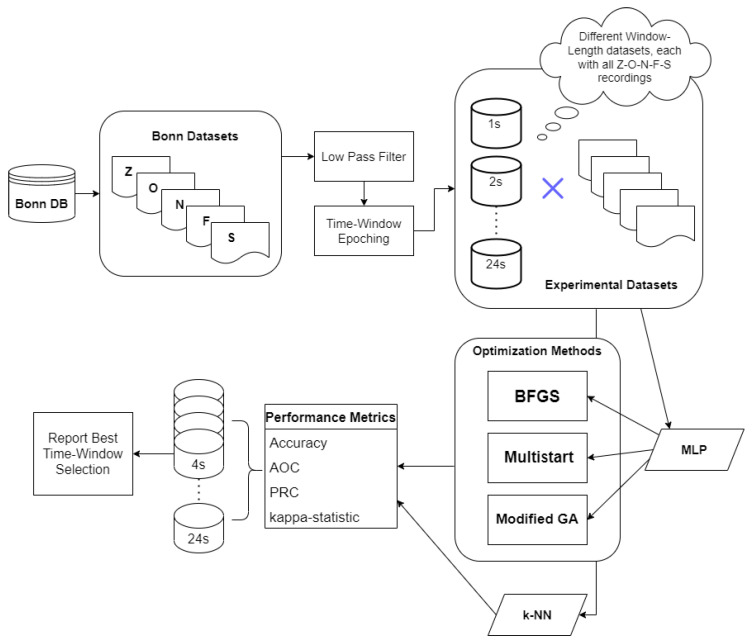
Flowchart of the proposed methodology.

The current research investigated the role of the window size in epilepsy EEG signal analysis by running a series of experiments using the database from the University of Bonn [19]. The tests were performed using a 10-fold cross-validation and are visualized in Table 1 and Figure 1.

All experiments were repeated 30 times with the window size ranging from 1 to 24 s. The number in each method’s cell represents the average classification accuracy of the test set for each window size (1–24 s). The accuracy for one fold was defined as the number of correctly classified instances divided by the total number of instances, as seen in Formula (12).

(12) $$accuracy=\frac{correctly\;classified\;instances}{total\;number\;of\;instances}$$

The accuracy was calculated by estimating the average value over all folds and then calculating the average value over all experiment runs. The SLNN used for training in the Broyden–Fletcher–Goldfarb–Shanno (BFGS), multistart and modified genetic algorithm (GA) methods had ten hidden neurons, and in every iteration of the multistart approach, a BFGS method was used to optimize the weights. Finally, the k-nearest neighbours (K-NN) method with K=2 was used.

In the experimental results depicted in Table 1, the bold fonts describe the time window that achieved the highest accuracy for each methodology.

It is seen that the window size dramatically impacted the accuracy values since when the window had a size of 20–21 s, the accuracy had its highest value and decreased when the window size gradually increased or decreased. The multistart method obtained the highest accuracy with a window size between 20 and 21 s (81.59%). Regarding the BFGS algorithm, the highest accuracy was achieved at with 20-s and 21-s time windows (80.92%), while the GA methodology achieved the highest accuracy when the time window was 21 s (81.06%). Finally, the K-NN algorithm achieved its best accuracy scores with a 22-s time window (81.17%).

Table 2 illustrates other standard evaluation measures for the K-NN algorithm, namely the area under the ROC, the area under the PRC, and the kappa statistic. The results of this table are in agreement with Table 1, with the 20–21-second time windows achieving the best performances at every evaluation metric.

## 5. Discussion

The current article investigated the time window size’s impact on EEG signal classification for epilepsy detection. The experimental part utilized three neural networks trained using three different algorithms (BFGS, multistart, modified GA) and the K-NN classifier. The experiments were repeated 30 times, and the average classification accuracy was reported.

The primary outcome from the experimental results summarized in Table 1 was that the window size in epilepsy EEG signals significantly impacted the classification accuracy of the compared methods. It was shown that for more accurate results, the window size must be between 20 and 21 s. Another significant outcome was the mixed results regarding the method which managed to get the best accuracy for each window size. There was no clear winning method for all window sizes, but the results varied when the window size changed.

An appropriate window length selection is crucial for machine learning methodologies on signal data (such as EEG). Too small time windows may fail to capture each condition’s signal characteristics. For example, a very small time window in an epilepsy methodology may result in not being able to capture the complete seizure waveforms. On the other side, too large time windows may capture signal properties of two different situations (such as ictal state and interictal state), thus negatively affecting the classification performance. The proposed study can be utilized in future methodologies that propose a classification scheme for EEG epilepsy detection problems. Our study’s resulting optimal window length agreed with another study proposed by Tzimourta et al. [53]. This study evaluated the optimal window length using different classification algorithms (naive Bayes, MLP, support vector machines, and decision trees) and found that 21-s windows achieved the best accuracy results. Moreover, our results suggested that the 20–21-s windows achieved the best performance. These findings agreed with Thangavel et al. [54], who classified epileptic signals using different features and examined different window lengths, concluding that the 20-s time window generated some of the best performance results.

However, some limitations regarding our methodology should be mentioned. One of them is the restricted length of the recordings, which did not allow exploring time windows larger than 24 s. To alleviate this limitation, a future extension of this methodology that incorporates longer EEG recordings from other publicly available databases should be performed. Furthermore, no wavelet transformations were used for the feature extraction step, as well as a limited number of machine learning algorithms were used (neural networks and K-NN), limiting the ability to generalize these findings to all automatic EEG epilepsy detection methodologies.

## 6. Conclusions

Epilepsy has attracted much attention from the research community because it can affect various people ranging from very young to the elderly. It can also have a serious economic impact on healthcare needs; it can cause premature deaths and lead to lost work productivity. Consequently, much scientific effort has been made to propose machine learning methodologies that perform automatic epilepsy detection from EEG signals. These methodologies commonly perform epoching of the time signals to produce the experiment’s training and test set. Thus, the window size in the signal decomposition is significant for detecting subtle changes in the EEG recording. This study evaluated the optimal time window length for four classification algorithms: three neural networks trained using the BFGS, multi-start and modified GA methods and the K-NN approach. Time windows from 1 to 24 s were explored and examined regarding the classification accuracy of the four algorithms. The epoching of 20–21 s achieved the best classification performance.

## Figures and Tables

**Table 1:** Experimental Results expressed in classification accuracy for the four algorithms employed regarding time windows ranging from 1 to 24 s. BFGS stands for Broyden–Fletcher–Goldfarb–Shanno algorithm. GA stands for genetic algorithm, K-NN stands for k-nearest neighbours.

Epoch (s)	BFGS	Multistart	GA	K-NN
1 s	56.86%	57.68%	56.91%	68.9%
2 s	65.06%	65.56%	65.06%	75.14%
3 s	69.7%	69.57%	69.01%	76.66%
4 s	72.62%	70.53%	70.06%	76.99%
5 s	75.69%	73.46%	71.96%	77.89%
6 s	74.63%	76.37%	75.44%	79.53%
7 s	74.76%	75.84%	74.43%	79.1%
8 s	76.06%	75.55%	74.95%	78.41%
9 s	76.25%	77.64%	76.5%	79.88%
10 s	76.96%	77.12%	76.38%	80.05%
11 s	76.42%	79.01%	77.2%	79.08%
12 s	76.55%	78.26%	77.06%	79.84%
13 s	77.04%	78.04%	76.05%	78.56%
14 s	77.81%	78.26%	77.13%	79.01%
15 s	79.75%	78.98%	78.41%	78.68%
16 s	77.35%	80.98%	78.59%	79.52%
17 s	77.7%	78.05%	77.82%	79.92%
18 s	78.5%	79.24%	78.10%	79.92%
19 s	80.7%	79.71%	78.47%	79.49%
20 s	**80.92%**	**81.59%**	80.78%	80.00%
21 s	**80.92%**	81.23%	**81.06%**	79.25%
22 s	80.04%	80.88%	81.00%	**81.17%**
23 s	80.69%	80.88%	80.89%	78.88%
24 s	80.25%	80.43%	79.98%	79.04%

**Table 2:** Area under the ROC, area under the PRC, and kappa statistic regarding the classification performance of the K-NN algorithm.

Epoch (s)	AOC	PRC	k-Stat
1 s	78.91%	48.6%	62.21%
2 s	79.89%	50.2%	68.74%
3 s	80.68%	50.1%	75.23%
4 s	86.44%	53.3%	71.95%
5 s	85.92%	56.8%	74.62%
6 s	85.45%	54.0%	76.38%
7 s	83.21%	58.1%	77.55%
8 s	87.21%	60.9%	77.19%
9 s	87.17%	61.8%	80.02%
10 s	86.57%	64.3%	78.84%
11 s	90.89%	64.2%	83.40%
12 s	90.49%	64.8%	82.32%
13 s	89.04%	68.1%	82.14%
14 s	88.88%	68.3%	82.85%
15 s	86.22%	70.4%	79.94%
16 s	85.45%	70.1%	80.15%
17 s	85.92%	73.6%	82.15%
18 s	84.70%	73.0%	84.29%
19 s	86.07%	74.7%	85.42%
20 s	92.22%	**78.5%**	**85.49**%
21 s	**92.51%**	76.5%	83.26%
22 s	88.70%	77.3%	82.44%
23 s	82.28%	75.7%	83.51%
24 s	88.37%	73.7%	80.00%

## Data Availability

The research utilizes the database from the University of Bonn [19].

## Abbreviations

The following abbreviations are used in this manuscript:

EEG	electroencephalogram
K-NN	k-nearest neighbours
BFGS	Broyden–Fletcher–Goldfarb–Shanno
SLNN	single-layer neural network
GA	genetic algorithm
BCI	brain–computer interface
MLP	multilayer perceptron
t-f	time frequency
PSD	power spectrum density
ANNs	artificial neural networks
CEEMDAN	complete ensemble empirical mode decomposition with adaptive noise
LPBoost	linear programming boosting
DWT	discrete wavelet transform
MODWT	maximal overlap discrete wavelet transform
PE	permutation entropy
SVM	support vector machine
CSI	combined seizure index
PCA	principal component analysis
